# The unsatisfactory state of physical activity and lifestyle among schoolchildren calls for changes in physical education teacher training

**DOI:** 10.3389/fspor.2026.1852327

**Published:** 2026-07-31

**Authors:** Dorota Groffik, Karel Frömel, Mateusz Ziemba, Michal Vorlíček, Josef Mitáš

**Affiliations:** 1 Faculty of Physical Education, Academy of Physical Education in Katowice, Katowice, Poland; 2 Faculty of Physical Culture, Palacký University, Olomouc, Czechia; 3 Chorzow Faculty, WSB Merito University in Poznań, Chorzow, Poland; 4 P. J. Šafárik University, Košice, Slovakia

**Keywords:** physical activity, physical literacy, steps per day, teacher training, wearables

## Abstract

**Background:**

Improving physical activity (PA) and lifestyle among schoolchildren has long been a priority in health policy; however, the outcomes of these global efforts remain unsatisfactory. One key prerequisite for achieving positive change is improving the professional training of physical education (PE) teachers. The study aimed to examine changes in PE teacher training focused on monitoring students' own PA, to compare these findings with evidence from PA research among schoolchildren, and support the development of health and physical literacy in future PE teachers.

**Methods:**

The study was conducted within the professional training of PE students from 2011 to 2022. A total of 472 men and 487 women participated in PA monitoring. Daily step counts were assessed using Yamax Digiwalker pedometers and Garmin Vívofit smart bands. Weekly PA, PA motivation, PA preferences, and well-being were assessed using the IPAQ–Long Form, motivation and preference questionnaires, and the WHO-5 Well-Being Index.

**Results:**

Participants were able to compare their PA monitoring results with the status and trends in PA among children and adolescents. Similar patterns were analyzed in gender differences, the structure of weekly PA, and the associations between PA, PA preferences, motivation for PA, and well-being. Participation in organized PA was associated with higher odds of meeting vigorous PA recommendations, increasing the likelihood of compliance by approximately 1.9 times.

**Conclusions:**

Integrating the verification of PA-related evidence through personal PA monitoring into the professional training of PE students creates favorable conditions for transferring PA knowledge into school practice. This approach may enhance support for PA, healthy lifestyles, and physical literacy among schoolchildren and school leadership.

## 1 Introduction

Promoting the health of school-aged children is closely linked to the promotion of physical activity (PA) and physical fitness. Despite substantial evidence documenting the health risks associated with insufficient PA in children and adolescents (1, 2), the overall status and trends in PA among young people have not improved (3). Physical activity is not sufficiently prioritized within adolescent health promotion strategies (4). Nevertheless, lifestyle behaviors adopted during adolescence represent a critical foundation for health in adulthood (5–7), as higher levels of PA in adolescence are associated with greater PA later in life (8). Participation in organized sport during childhood and adolescence is crucial, as it is associated with higher physical fitness in adulthood (9), higher overall weekly PA, and a greater likelihood of meeting PA recommendations (10). Organized youth sport participation is also linked to healthier lifestyle choices in adulthood (11). Similarly, participation in PE lessons is positively associated with a range of health-related behaviors among adolescents (12). Consequently, both school-based PE and out-of-school PA are considered important contributors to health across the life course (13).

These positive associations between organized PA participation and overall PA levels were substantially disrupted by the COVID-19 pandemic (hereafter the pandemic). Research has documented marked declines in children's and adolescents' participation in organized PA (14, 15), a global reduction in PA levels (2), and a decrease in compliance with PA recommendations (16, 17). The pandemic exerted an unprecedented negative impact on young people's living conditions and lifestyles (18). In Slovenia, this was reflected in the most significant decline in children's physical fitness observed over the past three decades (19). Importantly, restrictions on organized PA during the pandemic disrupted established PA habits (20), and many students were unable to compensate for the loss of structured activities through alternative forms of individually performed PA (21).

The pandemic also revealed insufficient preparedness among school leaders and teachers to respond to crisis-related restrictions in education. Schools were largely unprepared for distance education in PE (22) and for supporting PA among students confined in their homes (21). Inadequate online PA provision may reflect limited physical literacy, insufficient competence in selecting appropriate PA during crisis, or the historically limited effectiveness of online PE formats (23). Adolescents, in particular, appeared unprepared to engage in independent PA at home or to offset the demands of distance learning through adequate movement behavior (24). According to Kovács et al. (25), 81% of European students aged 6–18 years failed to meet the recommendation of at least 60 min of PA per day during the pandemic. Similarly, research in Czech and Polish schools showed that 88% of adolescents did not meet the recommendation of at least 60 min of moderate-to-vigorous PA (MVPA) on at least five days per week combined with at least 20 min of vigorous PA on three days per week (24).

Given the documented negative impact of the pandemic on children and adolescentś PA—and the possibility of future societal or public health crises—it is essential to identify the underlying causes of schools' insufficient preparedness for such situations. Even prior to the pandemic, limited PA among adolescents was associated with inadequate knowledge of PA recommendations. Marques et al. (26) reported that 43.5% of adolescents were unaware of the recommendation of 60 min of daily PA, and only 62.7% correctly identified recommendations related to PA intensity. Alarmingly, only 7.5% of PE teachers were able to correctly identify PA recommendations, and they generally lacked awareness of how well children and adolescents meet health-related PA guidelines (27).

Insufficient knowledge of PA recommendations has also been documented among university students, including those enrolled in sport and health-related programs (28, 29). In this context, the call by DiPietro et al. (30) to train new professionals capable of effectively translating PA and public health evidence into practice has become particularly relevant in the post-pandemic period. Consequently, addressing shortcomings revealed during the pandemic requires systematic changes in the professional training of future PE teachers (21).

Harris (31) critically evaluated the preparation of PE teachers to promote healthy and active lifestyles and emphasized the need for curriculum changes that better support PA promotion among adolescents. The importance of future educators' knowledge of health and PA has also been highlighted by Moreno-Lavaho et al. (32), who stress the need to consider gender differences and PA levels when designing PA education strategies. Supporting a healthy lifestyle among teachers is important not only for their own well-being but also because teachers serve as behavioral role models for their students (33). PE teachers, in particular, play a central role in fostering health literacy among educational professionals (34).

Relatively few studies have examined the role of PE teachers and other educators in research focusing on adolescents' PA and lifestyle behaviors. While some studies suggest that parents exert a more substantial influence on adolescents' PA than PE teachers (35), others report comparable impact (36). Nevertheless, PE teachers are widely perceived as key promoters of PA and healthy lifestyles among youth (37). Their influence, however, is shaped by national education and health policies, school systems, and the broader societal status of educators. In light of rapid lifestyle changes among school-aged youth, it is therefore crucial to enhance the quality of PE teacher training so that future teachers are equipped to anticipate and effectively address emerging barriers to PA and healthy living.

An essential component of such training is enabling future PE teachers to gain direct experience with PA monitoring, wearable technologies, and the practical application of PA recommendations. The importance of teachers' own experience in meeting PA recommendations has been emphasized even at the elementary school level (38), particularly given findings that only 23% of elementary school teachers meet the recommendation of 20 min of daily PA. Professional training grounded in evidence-based PA research may therefore facilitate a more effective translation of PA knowledge into school and extracurricular practice.

The present study is based on a socio-ecological model of PA and health promotion, emphasizing the role of key moderators of physical behavior within educational settings (39). It also draws on self-regulation theory (40), which helps explain the relationships between intentions, adherence to PA recommendations, and actual PA behavior. In comparing the performance-oriented PA patterns of PE students with the more diverse motivational profiles of adolescents, goal-setting theory in sport was also considered (41).

Based on these theoretical premises, the study addresses the following research questions:

- What are the levels and trends in PA among PE students during professional training?
- How does the PA of PE students differ from that of school-aged youth?
- To what extent should PA monitoring be incorporated into PE teacher education curricula?

The aim of the study is to examine changes in the professional training of PE teachers through the monitoring of their own PA, compare these findings with results from research on PA among school-aged youth, and support the development of health and physical literacy in future PE teachers.

## 2 Methods

### 2.1 Participants and procedure

The study was conducted between 2011 and 2022 within the professional training of future PE teachers at the Jerzy Kukuczka Academy of Physical Education in Katowice. A total of 472 men (mean age = 23.9 ± 1.4 years; mean body mass = 77.8 ± 9.6 kg; mean height = 180.1 ± 6.9 cm; mean BMI = 23.9 ± 2.2 kg·m⁻^2^) and 487 women (mean age = 23.1 ± 1.6 years; mean body mass = 59.9 ± 7.5 kg; mean height = 167.4 ± 6.1 cm; mean BMI = 21.3 ± 2.1 kg·m⁻^2^) participated in PA monitoring. Each year, approximately 50–90 PE students were included. Different students participated each year, and repetition of the research was not possible. Students repeating a year of study were not included in the research. Participants were enrolled either in the compulsory course *Diagnostics of Physical Activity of Children and Youth* or were involved in PA monitoring as part of their master's thesis research. As the study was embedded in PE teacher training, all eligible students enrolled in the course or holding ethics approval for school-based PA research were invited to participate. Participation was voluntary, and students could withdraw at any time and complete alternative academic tasks; however, no withdrawals occurred.

The study aimed to connect education and research in the professional training of PE teachers through support for PA and the use of wearables in both professional training and school practice; therefore, it had the character of a cross-sectional study. Replication of the study in the presented form is not possible because of social and educational changes over time; however, repeating the study under similar university conditions is both feasible and desirable.

Throughout the study period, data collection was supervised by the same three-member academic teaching team. Students were informed about the aims and practical benefits of the study, participated in group-based analyses of PA data, and received individual and group feedback on the results. At the beginning of the study, PE students registered in the web-based *International Database for Research and Educational Support* (Indares.com), which was used for data collection and educational feedback. Due to disruptions caused by pandemic-related restrictions, the study was organized into three four-year periods: 2011–2014 (121 men and 176 women), 2015–2018 (176 men and 157 women), and 2019–2022 (175 men and 154 women). In addition to the basic sample (*n* = 959), subsets were used for analyses of associations between step counts and motivation for PA (*n* = 912), and between step counts and well-being (*n* = 530).

### 2.2 Measures

#### 2.2.1 Physical activity monitoring

Weekly PA monitoring was conducted using Yamax Digiwalker SW-700 pedometers between 2011 and 2014 and Garmin Vívofit 1 and Vívofit 3 smart bands from 2015 to 2022. Previous validation studies have demonstrated no significant differences in average daily step counts between Yamax pedometers and Garmin Vívofit devices under free-living conditions (42, 43). Daily step counts were restricted to a valid range of 1,000–30,000 steps per day, consistent with previous research (44, 45). Inclusion criteria required at least three monitored school days and one weekend day. Missing school-day values were replaced using the mean of the remaining school days, while missing weekend values were replaced using the available weekend day. A total of 234 PE students were excluded due to non-compliance with the monitoring criteria, mainly because of exceeding 30,000 steps per day or recording a smaller number of monitoring days. Missing data for the required number of days were completed for 321 participants. This approach is based on the evaluation of daily step-count records in similar studies (45). Thus, 959 participants were included in the basic set. Participants wore the monitoring devices continuously for one week, excluding the first familiarization day, from morning (after personal hygiene) until bedtime.

#### 2.2.2 Questionnaires

Weekly PA levels and PA structure were assessed using the International Physical Activity Questionnaire–Long Form (IPAQ) (46), administered in its standardized and research-validated Polish version (10). The questionnaire captures PA across multiple domains (school-related, transportation, household, recreational, sport, and leisure-time PA), PA intensity (vigorous, moderate, walking), and sedentary behavior.

Motivation for PA was assessed using the Motives for Physical Activity Measure–Revised (MPAM-R), which includes 30 items across five dimensions: enjoyment, competence, appearance, fitness, and social motives (47). A total of 912 PE students met the inclusion criteria for analyses examining associations between PA and PA motivation.

Preferences for PA types were assessed using the Physical Activity Preference Questionnaire, which has been previously used in adolescent and young adult populations (48, 49). PE students selected their five most preferred PA types from predefined categories, including individual PA, team PA, fitness-related PA, water-based PA, outdoor PA, martial arts, rhythmic/dance PA, and overall PA. Preferences ranked from first to third place were included in the analyses.

Well-being was assessed using the Polish version of the WHO-5 Well-Being Index, a validated instrument suitable for population-based research (50). Participants were categorized according to established cut-off values: <13 points indicating lower well-being and ≥13 points indicating higher well-being. As the WHO-5 was introduced later into the Indares system, data were available for 530 participants.

All questionnaires were completed prior to PA monitoring in the following order: IPAQ, MPAM-R, WHO-5 Well-Being Index, and the PA preference questionnaire. Consequently, questionnaire-based assessments were not influenced by subsequent PA monitoring.

#### 2.2.3 Physical activity recommendations

Daily step-count recommendations were defined according to established pedometer-based thresholds for adolescents, using a criterion of 11,000 steps per day (51, 52). Subjective PA recommendations derived from IPAQ data were adapted from Healthy People 2030 and the Physical Activity Guidelines for Americans (53, 54).

The following PA recommendations were applied:

- at least 3 × 20 min of vigorous PA,
- at least 5 × 30 min of walking PA,
- at least 5 × 60 min of MVPA,
- a combined recommendation of at least 5 × 60 min of MVPA and 3 × 20 min of vigorous PA.

The reason for establishing these PA recommendations, which are typically used for adolescents, was to simplify comparative analyses between the PA of PE students and adolescents. This also made possible to better understand the advantages and disadvantages of using PA recommendations in practice.

### 2.3 Statistical analysis

Statistical analyses were performed using Statistica version 13 and IBM SPSS Statistics version 25. Descriptive statistics and the Kolmogorov–Smirnov and Lilliefors tests were used to assess data distributions. Differences by gender, day of the week, and study period were analyzed using repeated-measures analysis of variance (ANOVA) with Scheffé *post hoc* tests. Box's M test and Mauchly's test of sphericity were applied to evaluate ANOVA assumptions.

One-way ANOVA with Scheffé *post hoc* tests and the Kruskal–Wallis test were used to examine associations between PA and selected PA-related moderators. Group differences were additionally analyzed using the Mann–Whitney U test, and compliance with PA recommendations was examined using cross-tabulations. Binary logistic regression using the enter method was applied to estimate odds ratios (ORs) for meeting PA recommendations. Effect sizes were interpreted using partial eta squared (*η*_p_^2^) and effect-size coefficients (r) as follows: small (*η*_p_^2^ ≥ 0.01; r ≥ 0.10), medium (*η*_p_^2^ ≥ 0.06; r ≥ 0.30), and large (*η*_p_^2^ ≥ 0.14; r ≥ 0.50). Statistical significance was set at *p* < 0.05.

## 3 Results

### 3.1 Average physical activity of men and women by steps per day across the week

**Figure 1:**
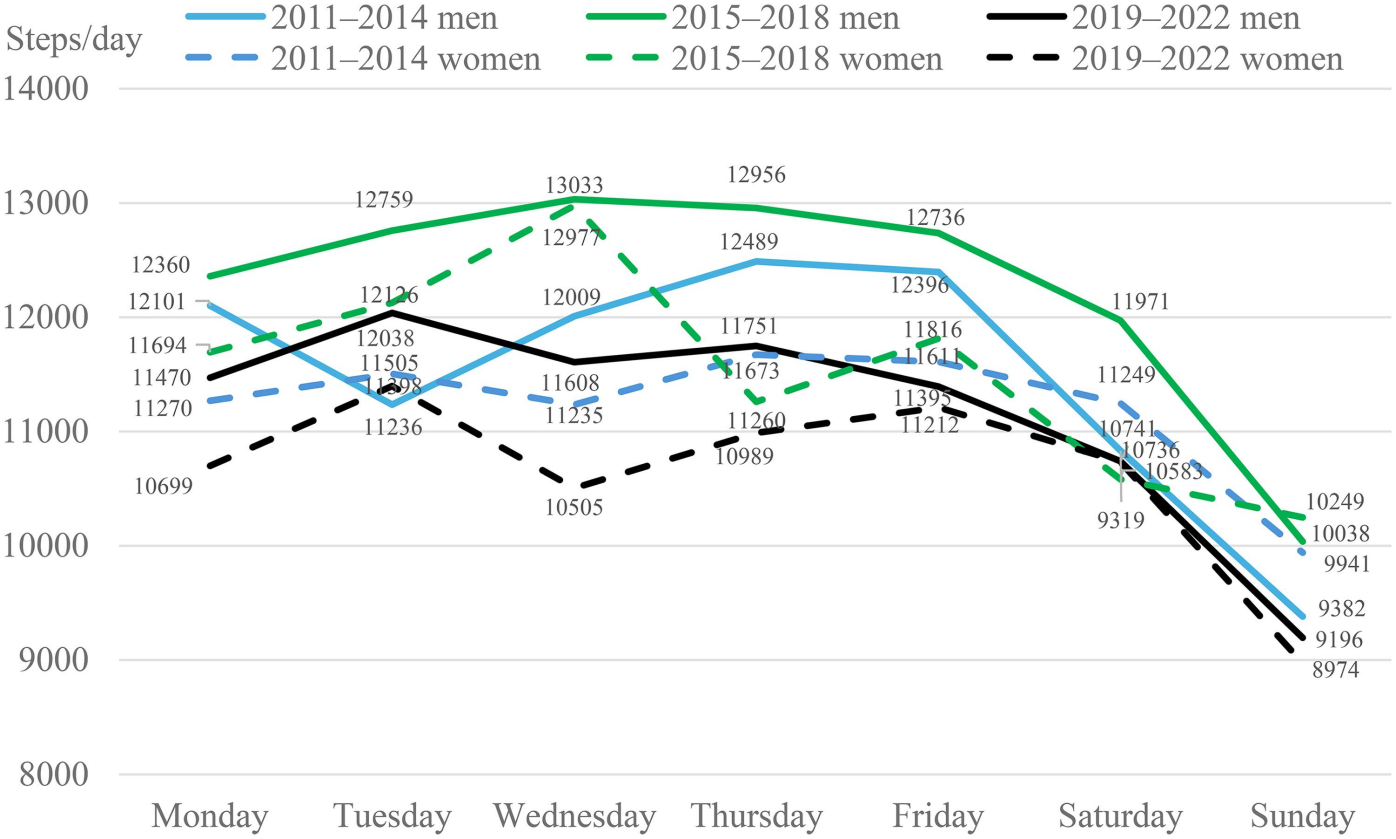
Weekly physical activity expressed as steps per day among men and women across individual days of the week, 2011–2022 (472 men and 487 women).

Repeated-measures ANOVA confirmed significant differences in men's steps per day across individual days of the week [F_(6, 2814)_ = 27.45, *p* < 0.001, *η*_p_^2^ = 0.055]. Step counts on Sundays were significantly lower than on all other days of the week (*p* < 0.001), and step counts on Saturdays were significantly lower than those on Wednesdays (*p* = 0.025) and Thursdays (*p* = 0.004) (Figure 1). The highest average value among men was observed on Wednesdays during the 2015–2018 period (13,033 steps per day), whereas the lowest was observed on Sundays during the 2019–2022 period (9,196 steps per day). The interaction between days of the week and study period was not statistically significant [Days × Period: F_(12, 2814)_ = 0.90, *p* = 0.897, *η*_p_^2^ = 0.004].

Among women, significant differences in steps per day were also observed across days of the week [F_(6, 2904)_ = 15.05, *p* < 0.001, *η*_p_^2^ = 0.030]. Women accumulated significantly fewer steps on Sundays than on all school days (*p* < 0.001) and Saturdays (*p* = 0.002). The highest average step count among women was recorded on Wednesdays during the 2015–2018 period (12,977 steps per day), while the lowest value was observed on Sundays in the 2019–2022 period (8,974 steps per day). A significant interaction between days of the week and study period was identified among women [Days × Period: F_(12, 2904)_ = 2.48, *p* = 0.003, *η*_p_^2^ = 0.010].

A significant interaction between gender and days of the week was also found [Days × Gender: F_(6, 5718)_ = 2.41, *p* = 0.025, *η*_p_^2^ = 0.006]. Men accumulated significantly more steps per day than women on Mondays (*p* = 0.028), Wednesdays (*p* = 0.047), Thursdays (*p* = 0.002), and across the average day of the week (*p* = 0.028).

Further analyses showed that higher motivation for PA among PE students was positively associated with higher average weekly step counts [F_(1, 910)_ = 5.20, *p* = 0.008, *η*_p_^2^ = 0.023]. Participation in organized PA was also associated with significantly higher average steps per day [F_(1, 957)_ = 7.01, *p* = 0.008, *η*_p_^2^ = 0.007]. No significant association was observed between overall well-being and average daily step counts [F_(1, 528)_ = 2.26, *p* = 0.134, *η*_p_^2^ = 0.004]. However, higher well-being was positively associated with higher step counts on school days [F_(1, 528)_ = 4.20, *p* = 0.041, *η*_p_^2^ = 0.008].

Preferences for specific types of PA were also associated with average weekly step counts. PE students who preferred fitness-related activities accumulated significantly more steps per day than those without this preference [F_(1, 957)_ = 3.90, *p* = 0.049, *η*_p_^2^ = 0.004; 11,710 ± 3,845 vs. 11,217 ± 3,627 steps per day]. Similarly, PE students preferring outdoor PA achieved higher step counts than those without this preference [F_(1, 957)_ = 7.97, *p* = 0.005, *η*_p_^2^ = 0.008; 11,862 ± 3,702 vs. 11,152 ± 3,696 steps per day]. On average, men accumulated significantly more steps on school days than women [12,170 vs. 11,468 steps per school day; F_(1, 953)_ = 6.94, *p* = 0.009, *η*_p_^2^ = 0.007]. Despite the statistical significance of the differences was found in daily step counts, some very low partial eta-squared values suggest that the observed associations explained only a minimal proportion of the variance in step counts.

### 3.2 Average weekly physical activity according to the international physical activity questionnaire

Based on IPAQ-derived estimates of weekly PA, significant gender differences were observed. Men reported significantly higher levels of vigorous PA (U = 5.25, *p* < 0.001, *η*^2^ = 0.058) and school-related PA (U = 3.50, *p* < 0.001, *η*^2^ = 0.026). In contrast, women reported significantly higher levels of transportation-related PA (U = 2.24, *p* = 0.025, *η*^2^ = 0.011) and household PA (U = 6.42, *p* < 0.001, *η*^2^ = 0.087). No significant gender differences were found in total weekly PA or between individual study periods [H_(5, 959)_ = 7.86, *p* = 0.164, *η*^2^ = 0.003].

### 3.3 Compliance with physical activity recommendations

**Figure 2:**
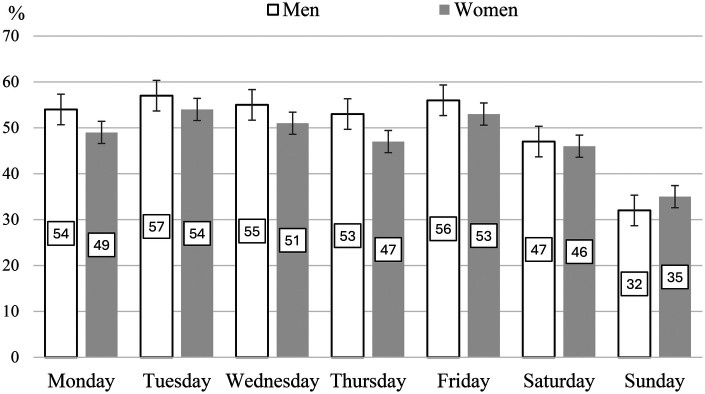
Compliance with the recommendation of 11,000 steps per day among men and women across individual days of the week (*n* = 959).

No significant gender differences were observed in compliance with the recommendation of 11,000 steps per day across individual days of the week, as assessed by wearables (Figure 2). For both men and women, compliance was lowest on Sundays and significantly lower than on all other days of the week (*p* < 0.001).

**Figure 3:**
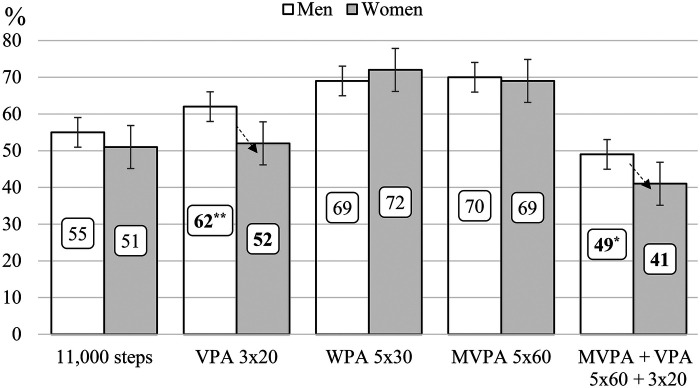
Compliance with physical activity recommendations: 11,000 steps per day; vigorous physical activity (VPA), 3 × 20 min; walking physical activity (WPA), 5 × 30 min; moderate-to-vigorous physical activity (MVPA), 5 × 60 min; combined MVPA + VPA recommendation (5 × 60 min of MVPA and 3 × 20 min of VPA). ^*p^ < 0.05; **p < 0.01.

Based on subjective assessment of PA, significant gender differences were found in compliance with vigorous PA recommendations (*χ*^2^ = 10.40, *p* = 0.001, r = 0.104) and with the combined recommendation for MVPA and vigorous PA (*χ*^2^ = 6.00, *p* = 0.014, r = 0.079) (Figure 3).

### 3.4 Odds of meeting physical activity recommendations by participation in organized physical activity

Participation in organized PA was associated with a higher likelihood of meeting the combined PA recommendation of at least 5 × 60 min of MVPA and 3 × 20 min of vigorous PA (Table 1). PE students participating in organized PA were approximately 1.9 times more likely to meet this combined recommendation than students not participating in organized PA.

**Table 1:** Odds ratios for meeting the physical activity recommendation (5 × 60 min of moderate-to-vigorous physical activity and 3 × 20 min of vigorous physical activity) according to participation in organized physical activity (*n* = 959).

Variables		Model 1		Model 2		Model 3	
		OR	*p*	OR	*p*	OR	*p*
		(95% CI)		(95% CI)		(95% CI)	
Organized PA	No ref.	1.99	<0.001	1.89	<0.001	1.93	<0.001
	Yes	(1.48–2.67)		(1.40–2.55)		(1.42–2.61)	
Gender	Men ref.			0.85	0.254	0.84	0.206
	Women			(0.65–1.12)		(0.63–1.10)	
Age (years)	<23			0.78	0.120	0.76	0.091
	23–26 ref.			(0.58–1.07)		(0.56–1.04)	
Body mass index	Normal			0.72	0.079	0.73	0.096
	Overweight ref.			(0.50–1.04)		(0.50–1.06)	
Smoking status	No ref.			0.92	0.749	0.92	0.726
	Yes			(0.57–1.50)		(0.56–1.49)	
City size (inhabitants)	<30,000					0.90	0.484
	≥30,000 ref.					(0.67–1.21)	
Car ownership	No ref.					0.89	0.645
	Yes					(0.65–1.20)	
Dog ownership	No					0.85	0.229
	Yes ref.					(0.64–1.11)	
Bicycle ownership	No ref.					0.99	0.961
	Yes					(0.71–1.38)	
Use of a weekend house	No ref.					0.98	0.957
	Yes					(0.68–1.41)	

OR, odds ratio; CI, confidence interval; PA, physical activity.

Model 1: unadjusted model including participation in organized PA (no, yes).

Model 2: adjusted for gender (men, women), age (<23 years, 23–26 years), body mass index (normal weight, overweight), and smoking status (no, yes).

Model 3: additionally adjusted for city size (<30,000, ≥30,000 inhabitants), car ownership (no, yes), dog ownership (no, yes), bicycle ownership (no, yes), and use of a weekend house (no, yes).

Adjustment for covariates in Model 2 (gender, age, body mass index, smoking status) and Model 3 (city size, car ownership, dog ownership, bicycle ownership, and weekend house use) did not alter the significance of this association.

### 3.5 Secondary effects of changes in physical education teacher training

Anonymous PE student evaluations consistently rated the course *Diagnostics of Physical Activity of Children and Youth* positively. The course reached its enrollment limit each year. Over the study period, interest among PE students in using wearables in school and extracurricular settings increased. As a result of increased interest, the number of wearable devices made available to students also increased. Experience gained through personal PA monitoring was reflected in increased interest in implementing monitoring technologies during school-based teaching practice. Successful use of wearables in PE lessons by preservice teachers further stimulated interest in innovative didactic approaches. The best PE students who passed the research also successfully participated in national competitions every year, aimed at promoting new approaches to PE lessons. Additionally, between 12 and 16 master's theses per year were defended using wearable-based PA monitoring in school settings.

## 4 Discussion

The primary contribution of this study is the presentation of a long-term, empirically verified approach to integrating PA monitoring into PE teacher training. By systematically monitoring and evaluating their own PA, PE students were able to verify theoretical knowledge and evidence-based findings through direct personal experience. Individual- and group-level analyses of PA outcomes were subsequently compared with PA patterns observed among children and adolescents, particularly in Polish school settings (55). This experiential approach appears to create favorable conditions for transferring of PA-related knowledge into both health-oriented practice (56) and school-based education (57). However, associations between PE teacher training and PA levels among school-aged youth remain insufficiently explored (58), and the conclusions of the present study therefore require further verification in school-based practice.

Gender differences in PA observed among PE students largely mirrored patterns reported in adolescent populations. Higher levels of vigorous PA among men and higher levels of walking among women are consistent with previous findings among school-aged youth (59). Importantly, total weekly PA did not differ significantly by gender when assessed by IPAQ, suggesting that gender differences may be more pronounced in PA intensity and specific PA domains than in overall PA volume. Awareness of these patterns during PE teacher training may support more sensitive and effective strategies to address gender disparities in PA participation among schoolchildren, particularly among girls (60).

Across the week, both male and female PE students accumulated the lowest levels of PA on Sundays, a pattern consistently documented among children (61), adolescents (62), and older adults (63). Awareness of these temporal distributions of PA may help future PE teachers design targeted interventions for days with lower activity levels. From an educational perspective, such insights are particularly relevant for structuring school-based and extracurricular PA opportunities throughout the week.

Unlike findings among children and adolescents, higher well-being among PE students was not associated with higher average daily step counts (64, 65). However, positive associations were observed between well-being and PA accumulated on school days. Previous research has shown that vigorous PA is positively associated with adolescent well-being (66), underscoring the importance of structured and sufficiently intensive PA for mental health.

Participation in organized PA emerged as a key factor associated with higher levels of vigorous PA among PE students, consistent with evidence from adolescent populations (60, 67). Experiencing these associations firsthand may enhance future teachers' understanding of the importance of organized PA in supporting health-enhancing behaviors. In addition, congruence between preferred and practiced PA was associated with higher PA levels, highlighting the importance of respecting students' PA preferences when promoting participation in school and community settings (67).

Wearable technologies and digital tools for PA monitoring are increasingly important for PA promotion and health education (68). Their integration into PE teacher training offers opportunities to develop practical competencies related to PA assessment, data interpretation, and individualized feedback. The findings of this study suggest that at least one week of wearable-based PA monitoring represents the minimum exposure required for students to understand both the benefits and limitations of these technologies. Familiarity with commonly used wearable devices and awareness of their measurement constraints should therefore be considered essential components of contemporary PE teacher education (69, 70).

A particularly important area for improvement in PE teacher training concerns comprehensive knowledge of PA recommendations and practical experience in implementing them. Knowledge of PA guidelines is a core component of health and physical literacy, and personal experience with meeting PA recommendations may enhance teachers' credibility and confidence when promoting PA among students (27). Previous studies have identified substantial gaps in PE teachers' knowledge of PA recommendations (71, 72), highlighting the need to address this issue systematically during initial teacher education and continuing professional development.

PE teachers occupy a unique position at the intersection of education, health promotion, and public health. Consequently, elements of broader professional training—beyond pedagogical instruction alone—remain highly relevant, particularly when preparing teachers to interpret evidence-based PA recommendations and to communicate both their benefits and limitations. This includes understanding the relationship between PA recommendations and health outcomes such as mortality and cardiovascular disease (73), as well as the appropriateness of performance-oriented goals in educational settings (74, 75).

Importantly, PA promotion in school and out-of-school settings should not exacerbate inequalities between more and less physically active adolescents. Adequate PA support must be characterized by equity, inclusiveness, and equal access to organized PA opportunities (76). PE teachers should therefore be equipped to adopt balanced and context-sensitive approaches to PA promotion that consider individual abilities, motivations, and social environments.

Strengthening the integration of PA theory, practice, and research into PE teacher training may expand the role of PE teachers beyond the school setting and position them as key contributors to public health initiatives (77, 78). Such training may also support the development of online and hybrid PE formats, more effective use of wearable technologies, and cross-curricular integration of health-related topics.

The present findings also support the inclusion of short-term PA monitoring using wearables as a standard component of professional education in health-related fields. Similar experiential approaches have demonstrated benefits in the professional training of physiotherapists, suggesting that PA monitoring may represent a transferable educational strategy across health-related disciplines (79).

Several limitations of the study should be acknowledged. The sample consisted of PE students who were inherently interested in PA, which may limit generalizability. In addition, participation in PA monitoring was embedded within course requirements and thesis-related research, which may have influenced engagement levels. Given the demanding nature of PA monitoring using wearables, it was not possible to conduct the study simultaneously at other institutions in Poland. Only parallel PA monitoring of Czech physiotherapy students proved beneficial (79). Another limitation of the study is the change in the use of wearables for PA monitoring influenced by technological development. The subjective assessment of PA types using the IPAQ is also a limitation of the study. However, comparing these results with wearable-based monitoring enabled a better understanding of the advantages and disadvantages of PA assessment. The study also did not allow for an objective evaluation of how effectively the acquired knowledge and experience related to PA monitoring will be applied in later professional practice. Future longitudinal research should therefore examine the long-term effect of PA monitoring during teacher education on teaching practices and PA outcomes among school-aged youth.

## 5 Conclusion

This study presents twelve years of experience with integrating PA monitoring into PE teacher training. Through systematic monitoring and evaluation of their own PA, PE students were able to verify evidence-based knowledge about PA and gain practical experience with the use of wearables in educational settings. PE students also had the opportunity to analyze the positive and negative associations between motivation for PA, weekly PA levels, and achievement of PA recommendations. Comparing PA patterns observed during teacher education with findings from research on PE of children and adolescents could positively influence the transfer of PA-related knowledge into school and out-of-school practice. The proposed and empirically verified changes in PE teacher training aim to strengthen the role of future PE teachers not only within schools but also among other educators and stakeholders involved in health promotion. However, the reported secondary effects of changes in PE teacher training still require longer-term monitoring in school practice. By supporting the development of health and physical literacy among students, parents, and educators, these changes may contribute to more effective promotion of physically active and healthy lifestyles among school-aged youth.

## Data Availability

The data are available at the following link: https://zenodo.org/records/21453788.
